# DAILY - A Personalized Circadian Zeitgeber Therapy as an Adjunctive Treatment for Alcohol Use Disorder Patients

**DOI:** 10.1192/j.eurpsy.2023.717

**Published:** 2023-07-19

**Authors:** N. Springer, L. Echtler, J. Hochenbleicher, E. Hoch, G. Koller, A. Hühne-Landgraf, D. Landgraf

**Affiliations:** ^1^Psychiatry, Ludwig-Maximilians-Universität München; ^2^IFT Institut für Therapieforschung, Munich, Germany

## Abstract

**Introduction:**

Hallmarks of alcohol use disorder (AUD) are disturbances of circadian rhythms and everyday structures. While circadian rhythms dictate the timing of daily recurring activities such as sleep, activity, and meals, conversely, these activities represent time cues, so called *Zeitgebers*, that the circadian system uses to synchronize with the environment. We have developed a novel therapy approach for AUD patients (DAILY), in which we take advantage of this mutual influence and stabilize and strengthen their circadian system by creating strict schedules for daily *Zeitgeber* activities (Hühne *et al.* Front Psychiatry 2021). Since every person has a circadian system with its own characteristics and is subject to social obligations, the daily plans are personalized for each test person.

**Objectives:**

We investigated whether the DAILY intervention can serve to increase the success of standard psychotherapy service and reduce alcohol use and relapse in AUD patients who are currently undergoing qualitative detoxification or post-detoxification therapy and are highly vulnerable to relapse at this stage. In addition, we investigated whether possible depressive symptomatology, sleep quality, and physical recovery of the participants are improved.

**Methods:**

In a 6-week controlled, randomized, single-blinded, parallel-group intervention study, we used detailed, 14-day diary entries to determine the optimal eating and sleeping times for each participant individually and used these to create personalized daily structure plans. Intervention participants were encouraged to adhere strictly to this plan for the following four weeks, with compliance verifiable by continuing the diary. Relapses and dropouts were documented, and questionnaires on mood state and sleep quality were completed at the beginning and end of the intervention. The control group received a sham treatment with no effect on their daily structure.

**Results:**

Our data show that DAILY therapy significantly improves meal and sleep time regularity and significantly reduces relapse rates, with 60% relapse rate in the control group and 11% in the intervention group (Figure 1A). In addition, the data show that among the few intervention participants who had relapses, these occurred on significantly fewer days during the study period than among relapsing control participants (Figure 1B). While depressive symptoms were unaffected by the DAILY therapy, sleep quality improved significantly (Figure 2).

**Image:**

**Figure:**
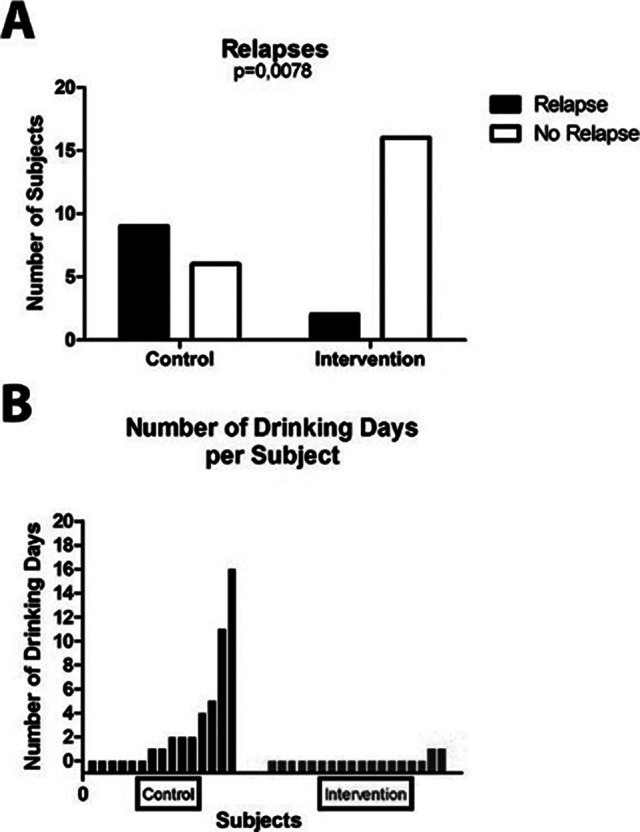

**Image 2:**

**Figure:**
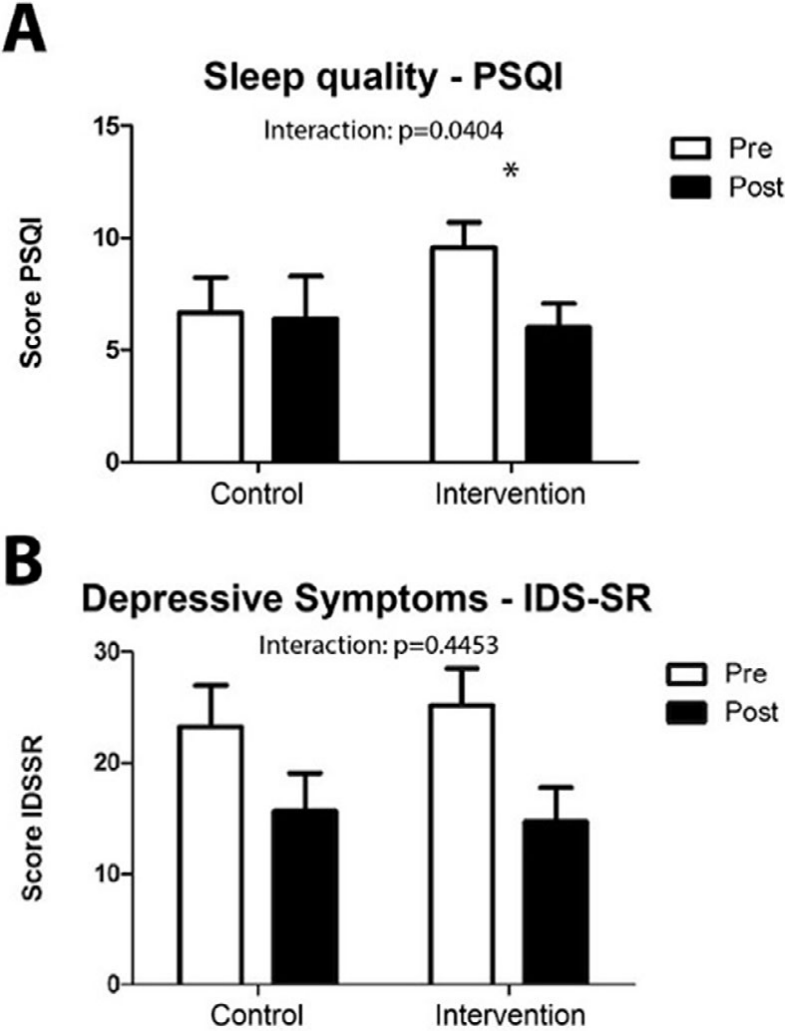

**Conclusions:**

Our data show that increasing the regularity of individual eating and sleeping times with DAILY is an effective tool to substantially promote abstinence during a period when AUD patients are particularly vulnerable to alcohol use and relapse, and in addition, to improve their sleep quality and accelerate physical recovery during therapy.

**Disclosure of Interest:**

None Declared

